# Defining Optimally Safe and Effective Blood Levels of Hydroxychloroquine in Lupus: An Important Step Toward Precision Drug Monitoring

**DOI:** 10.1002/art.70010

**Published:** 2026-02-17

**Authors:** Shivani Garg, Benoît Blanchet, Yann Nguyen, Fauzia Hollnagel, Ada Clarke, Michelle Petri, Murray B. Urowitz, John G. Hanly, Caroline Gordon, Sang‐Cheol Bae, Juanita Romero‐Diaz, Jorge Sanchez‐Guerrero, Ann E. Clarke, Sasha Bernatsky, Daniel J. Wallace, David A. Isenberg, Anisur Rahman, Joan T. Merrill, Paul R. Fortin, Dafna D. Gladman, Ian N. Bruce, Ellen M. Ginzler, Mary Anne Dooley, Rosalind Ramsey‐Goldman, Susan Manzi, Andreas Jönsen, Graciela S. Alarcón, Ronald F. Van Vollenhoven, Cynthia Aranow, Véronique Le Guern, Meggan Mackay, Guillermo Ruiz‐Irastorza, S. Sam Lim, Murat Inanc, Kenneth C. Kalunian, Søren Jacobsen, Christine A. Peschken, Diane L. Kamen, Anca Askanase, Jill Buyon, Julie Chezel, Alicja Puszkiel, Nathalie Costedoat‐Chalumeau

**Affiliations:** ^1^ University of Wisconsin School of Medicine and Public Health Madison; ^2^ Yale University New Haven, Connecticut; ^3^ Biologie du Médicament‐Toxicologie, Hôpital Cochin, Institut du Cancer Paris CARPEM, AP‐HP Centre and Université de Paris, CNRS, INSERM, CiTCoM, U1268 Paris France; ^4^ National Referral Centre for Rare Autoimmune and Systemic Diseases, Hôpital Cochin, AP‐HP Centre and Université Paris Cité and Centre de Recherche en Epidémiologie et Statistiques, Unité Inserm 1153, Université de Paris Cité Paris France; ^5^ Johns Hopkins University School of Medicine Baltimore Maryland; ^6^ Division of Rheumatology, University of Toronto and Schroeder Arthritis Institute, Krembil Research Institute, Toronto Western Hospital Toronto Ontario Canada; ^7^ Queen Elizabeth II Health Sciences Centre and Dalhousie University Halifax Nova Scotia Canada; ^8^ Department of Inflammation and Ageing, College of Medicine and Health University of Birmingham Birmingham United Kingdom; ^9^ Hanyang University Hospital for Rheumatic Diseases, Hanyang University Institute for Rheumatology, and Hanyang Institute of Bioscience and Biotechnology Seoul Korea; ^10^ Instituto Nacional de Ciencias Médicas y Nutrición Mexico City Mexico; ^11^ Mount Sinai Hospital and University Health Network, University of Toronto Toronto Ontario Canada; ^12^ Cumming School of Medicine University of Calgary Calgary Alberta Canada; ^13^ McGill University Health Centre Montreal Quebec Canada; ^14^ University of California Los Angeles; ^15^ University College London London United Kingdom; ^16^ Oklahoma Medical Research Foundation Oklahoma City; ^17^ Centre ARThrite‐UL, CHU de Québec–Université Laval Quebec City Quebec Canada; ^18^ Centre for Musculoskeletal Research, The University of Manchester, Manchester Academic Health Science Centre Manchester United Kingdom; ^19^ Centre for Public Health, Faculty of Medicine, Health and Life Sciences, Queen's University Belfast Belfast Northern Ireland United Kingdom; ^20^ State University of New York Downstate Medical Center Brooklyn New York City; ^21^ Thurston Arthritis Research Center, University of North Carolina at Chapel Hill; ^22^ Northwestern University Feinberg School of Medicine Chicago Illinois; ^23^ Allegheny Health Network Pittsburgh Pennsylvania; ^24^ Lund University Lund Sweden; ^25^ University of Alabama at Birmingham Marnix E. Heersink School of Medicine; ^26^ University of Amsterdam Amsterdam the Netherlands; ^27^ Feinstein Institute for Medical Research Manhasset New York; ^28^ Biobizkaia Health Research Institute, University of the Basque Country Barakaldo Spain; ^29^ Emory University School of Medicine Atlanta Georgia; ^30^ Istanbul University Istanbul Turkey; ^31^ University of California San Diego School of Medicine La Jolla; ^32^ Rigshospitalet, Copenhagen University Hospital Copenhagen Denmark; ^33^ University of Manitoba Winnipeg Manitoba Canada; ^34^ Medical University of South Carolina Charleston; ^35^ Hospital for Joint Diseases and Seligman Centre for Advanced Therapeutics, New York University New York City; ^36^ New York University School of Medicine New York City; ^37^ Bichat University Hospital, AP‐HP Paris France; ^38^ Biologie du Médicament – Toxicologie, Cochin University Hospital, AP‐HP and Université Paris Cité, INSERM, Optimisation thérapeutique en neuropharmacologie U1144 Paris France

## Abstract

**Objective:**

Using a hydroxychloroquine (HCQ) dose of 5 mg/kg/day in systemic lupus erythematosus (SLE) is associated with a higher risk of flares; HCQ blood level monitoring could be a better way to adjust the HCQ dose. We studied the upper threshold for a reference range of HCQ levels to inform routine monitoring.

**Methods:**

This observational study included patients (N = 2,010) across the Systemic Lupus International Collaborating Clinics, Wisconsin, international, and French studies who underwent HCQ blood level measurements. Using adjusted spline and logistic regression analyses on the cross‐sectional data, we first identified an HCQ blood level associated with higher HCQ toxicity. Next, we tested if this upper threshold level was supratherapeutic (no further risk reduction for the Systemic Lupus Erythematosus Disease Activity Index 2000 [score ≥6]). Finally, we examined associations between chronic kidney disease (CKD) stage and supratherapeutic (toxic) HCQ blood levels.

**Results:**

Among 1,842 patients (excluding 168 patients with very low HCQ blood levels), 4.9% had HCQ‐related toxicity. Odds of toxicity were 2.1‐fold higher with blood levels ≥1,150 ng/mL and 1.7‐fold higher with the cumulative HCQ dose per 1,000‐g increase. Blood levels ≥1,150 ng/mL were associated with a saturation in therapeutic effect, indicating supratherapeutic levels. Patients with CKD stage ≥3 had 2.3‐fold higher odds of having supratherapeutic levels (≥1,150 ng/mL).

**Conclusion:**

The therapeutic reference range for HCQ blood level monitoring is 750 to <1,150 ng/mL. HCQ level monitoring could optimize HCQ use, particularly in patients with CKD stage ≥3. Future longitudinal studies are needed to validate the use of HCQ blood level monitoring in optimizing dosing.

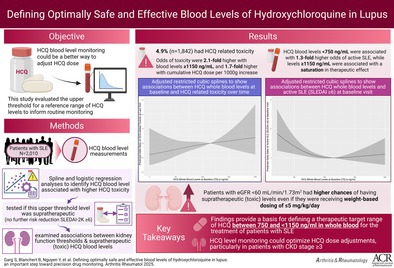

## INTRODUCTION

Hydroxychloroquine (HCQ) is a foundational therapy in the systemic lupus erythematosus (SLE or lupus) therapeutic armamentarium, as it prolongs disease‐free and damage‐free survival in patients.^1^ However, committing a patient to long‐term HCQ use can be challenging due to concerns, although rare, of eye and cardiac toxicity. Concerns for irreversible eye or cardiac toxicity exacerbate patient fears, leading to early discontinuation of medicine and nonadherence, which increases the risk of lupus flares and hospitalizations.^2^, ^3^, ^4^ Moreover, clinicians’ and patients’ concerns for toxicity are further amplified in patients with kidney disease given that over 60% of HCQ is cleared by the kidneys and HCQ is primarily dosed based on body weight.^2^, ^5^ Without clear guidance on adjusting HCQ doses in patients with chronic kidney disease (CKD), doses are either not adjusted or arbitrarily reduced, which could accelerate toxicity risk or increase the risk of SLE flares.^4^, ^6^, ^7^ Balancing efficacy and toxicity for HCQ is particularly difficult, especially amid an ongoing debate regarding the optimal dose.^8^, ^9^, ^10^

The conundrum to identify an optimal HCQ dose that minimizes harms and maximizes efficacy could be addressed by therapeutic HCQ blood level monitoring, which bypasses clinical variables affecting HCQ absorption or clearance and could guide optimal HCQ dosing in SLE.^10^, ^11^, ^12^, ^13^, ^14^, ^15^ Studies, including a global meta‐analysis, have established the clinical significance of using HCQ blood levels as an objective measure to monitor severe nonadherence and also clinical efficacy (lower risk of active lupus or flares).^10^, ^11^, ^12^, ^13^, ^14^, ^15^, ^16^, ^17^, ^18^ The proposed cutoffs to monitor for clinical efficacy are 750 and 1,000 ng/mL.^11^, ^16^, ^17^, ^18^ These cutoffs have a 96% negative predictive value (NPV) for active lupus with levels at or above these thresholds. However, cutoffs for HCQ blood levels associated with higher toxicity risk need further elucidation, as the published literature is conflicted. For instance, one population‐based cohort study reported significant associations between HCQ whole blood level tertiles (>1,183 ng/mL) and eye toxicity, whereas another cohort study of more adherent patients noted no associations between blood levels and systemic toxicity risk.^19^, ^20^ Clarity on HCQ whole blood levels associated with toxicity and the saturation of therapeutic effect, defined as no further clinical benefits in lowering SLE activity, is needed. These data are vital to inform the upper threshold of the therapeutic reference range for HCQ blood level monitoring to guide optimal HCQ use in SLE, balancing efficacy versus safety, delaying toxicity, and potentially alleviating patient worries regarding toxicity.^21^ Finally, having a defined therapeutic range for HCQ whole blood levels could enhance clinical uptake and guide clinicians with precise HCQ dosing based on individual patient risk factors, such as CKD.

In this study, we leveraged cross‐sectional data from diverse cohorts—including one cohort from the United States, data from two French studies and one international prospective study centralized in France, and data from a multicenter lupus cohort, the Systemic Lupus International Collaborating Clinics (SLICC) Inception Cohort—to clarify the upper threshold of HCQ blood levels associated with toxicity. Finally, we used recurrent visit data from the US cohort to examine the thresholds of kidney function associated with supratherapeutic HCQ levels to guide safe HCQ dosing in patients with lupus and CKD.

## PATIENTS AND METHODS

### Population

Cross‐sectional data were pooled from different sources: three cross‐sectional studies (n = 1,081), one longitudinal registry (n = 269), and one multinational cohort (n = 660) for a total of 2,010 patients. Three previously published cross‐sectional studies (n = 1,081) were centralized in Paris, France, and measured HCQ whole blood levels. These three studies included 203, 573, and 305 individuals from a single‐center study conducted in France, a multicentric study conducted in France, and a multicentric international study, respectively.^17^, ^18^, ^22^ A few patients could have been included in more than one study over the years, but the numbers were small.^17^, ^18^, ^22^ The next data source was a longitudinal registry from Madison, Wisconsin (institutional review board [IRB]: UW 2019‐0942), that included 269 patients with SLE. The final data source consisted of 660 patients from the SLICC Inception Cohort.^16^ Although the SLICC cohort was recruited between 1999 and 2011 from 33 centers in 11 countries within North America, Europe, and Asia (parent IRB: Toronto, IRB# 00‐0279),^23^, ^24^ HCQ serum levels were measured retrospectively at the laboratory of Cochin Hospital (Paris, France).^16^ For the current study, the validated published hematocrit‐based adjustment described by Blanchet et al was used to estimate whole blood concentrations from serum values.^16^, ^25^ Per the 2020 study, the ratio of serum to whole blood HCQ levels was 0.53 ± 0.15.^16^, ^25^ This method accounts for the distribution of analytes between plasma and red blood cells and has been shown to yield accurate and reproducible results across a range of hematocrit values. Therefore, for the current study, the validated 0.53 conversion factor was used to extrapolate equivalent levels of HCQ in whole blood from the serum levels in the SLICC cohort, similar to other studies.

Because we were interested in testing the upper limits of therapeutic blood levels, we excluded 168 patients with HCQ whole blood levels <200 ng/mL, as such very low levels have been associated with severe nonadherence,^16^ and included the remaining 1,842 patients in the main analysis. All patients (N = 2,010) were included in the sensitivity analyses.

### Variables

We abstracted key variables for each patient, including age, sex, and race or ethnicity, from the baseline or enrollment visit (T0). For the analysis, the weight‐based HCQ dose (T0) was categorized as ≤5 versus >5 mg/kg/day at the enrollment visit when HCQ blood levels (T0) were collected.^26^ Additionally, the duration of HCQ use was abstracted to calculate the cumulative HCQ dose (HCQ dose × duration of HCQ use). Cumulative HCQ dose was calculated until the last visit or the visit when HCQ‐related toxicity was noted (T_last visit_). Kidney function and the Systemic Lupus Erythematosus Disease Activity Index 2000 (SLEDAI‐2K) score were abstracted on the day of the baseline study visit (T0), when HCQ whole blood levels (T0) were measured. Kidney function (estimated glomerular filtration rate [eGFR]) was calculated using the 2021 Chronic Kidney Disease Epidemiology Collaboration equation. Active SLE on the study visit (T0) was defined as an SLEDAI‐2K score ≥6. Data on other variables such as steroid dose and other immunosuppressives at study visit (T0) were not uniformly available for all cohorts. HCQ levels from the baseline study visit (T0) were included and measured using a validated high‐performance liquid chromatography mass spectrometry assay. The assays were done in a research laboratory but are now commercially available through leading national laboratories (Exagen, Labcorp, ARUP, Mayo Clinic, Hopkins)^27^, ^28^ and covered by major payers under the *Current Procedural Terminology* (CPT) code 80020 in the United States. Again, as previously stated, HCQ levels measured in serum (SLICC cohort) were converted to whole blood levels using the following formula: HCQ serum levels/0.53.^16^ Finally, data on systemic irreversible HCQ toxicity (eg, eye [abnormal macular examination], heart, or muscle per biopsy findings; skin per clinical observation) over time (T_last visit_) were abstracted and categorized as present or absent. It is important to note that analyses included HCQ levels at T0 for all data sources, and HCQ levels at HCQ toxicity and the time to HCQ toxicity were not available. Only data on retinal toxicity were available for the SLICC cohort. One study (n = 305)^17^ did not have toxicity data. We performed a single condition imputation analysis using median HCQ toxicity incidence rates per our cohorts and literature.^29^, ^30^, ^31^, ^32^ This analysis revealed similar results when data from that single study were excluded.^17^, ^32^ Given similar results, we used the single imputation analysis for the final models to avoid sample size reduction.

### Primary analysis: HCQ blood levels (T0) and HCQ‐related toxicity (T_last visit_)

We used a validated three‐step approach to define the upper threshold for HCQ whole blood levels that are supratherapeutic and/or associated with a higher risk of systemic toxicity in 1,842 patients.^11^ First, we used the cut point analysis in R to identify an optimal HCQ blood level cutoff with a high NPV for toxicity. Additionally, the NPV of other cutoffs was tested in R as part of the sensitivity analysis. Next, we performed adjusted restricted cubic spline regression analysis to compare the estimated odds ratio (OR) of toxicity at higher HCQ blood levels (eg, 1,000 to <1,050 ng/mL, 1,050 to <1,100 ng/mL, 1,100 to <1,150 ng/mL, 1,150 to <1,200 ng/mL, 1,200 to <1,250 ng/mL, and 1,250 to <1,300 ng/mL) versus the upper cutoff for HCQ blood levels obtained from the optimal cut point analysis. This spline analysis informed the upper threshold for HCQ blood levels associated with higher toxicity odds. Finally, using multivariable logistic regression models, we tested different cutoffs for HCQ whole blood levels to identify the threshold significantly associated with higher toxicity risk. All models were adjusted for variables known to increase HCQ toxicity risk, including age, sex, cumulative HCQ dose, weight‐based HCQ dosing ≤5 versus >5 mg/kg/day, and eGFR. A separate sensitivity analysis was performed including all patients (N = 2,010). Finally, given heterogeneity between HCQ serum levels (data from SLICC cohort) and blood levels (other data sources), we performed a sensitivity analysis by separately analyzing associations in the SLICC cohort reporting HCQ serum levels and other data sources (US cohort and data from French studies) reporting HCQ whole blood levels.

### Secondary analysis: HCQ blood levels (T0) and active SLE (defined as SLEDAI‐2K score ≥6 at T0) to define supratherapeutic levels

Given low toxicity rates with HCQ, we completed optimal cut point, adjusted restricted spline regression, and multivariable logistic regression analyses to test if the upper threshold level was also supratherapeutic. Supratherapeutic levels were defined if levels beyond the upper threshold for HCQ blood levels led to no further change in odds of active SLE (a ceiling/saturation effect in response). All models were adjusted for variables known to increase active SLE risk, including age, sex, weight‐based HCQ dosing ≤5 versus >5 mg/kg/day, and eGFR. Finally, as described previously, additional sensitivity analyses were performed in all patients (N = 2,010) and separately in the SLICC cohort and data from other sources.

### Subgroup analysis to identify associations between weight‐based HCQ dose categories (≤5 and >5 mg/kg/day) at T0 and supratherapeutic levels (T0)

A plot was generated to summarize frequency of subtherapeutic, therapeutic, and supratherapeutic HCQ blood levels (T0) by weight‐based HCQ dose categories (T0). Next, we used the chi‐square test to check if counts were statistically significant and if HCQ level monitoring would be useful in the weight‐based dose categories.

### Identifying kidney function thresholds associated with supratherapeutic levels

Using data from all patients (n = 1,842), we first performed a restricted spline analysis to examine associations between HCQ blood levels (T0) and eGFR thresholds (T0), adjusting for age, sex, weight‐based HCQ dosing, and SLEDAI‐2K (T0). Next, significant eGFR thresholds were used in logistic regression analyses to estimate the odds of having supratherapeutic HCQ blood levels by eGFR thresholds (eg, >90 vs ≤90 mL/min/1.73 m^2^ or >60 vs ≤60 mL/min/1.73 m^2^). Additionally, regression plots were created to estimate changes in HCQ blood levels across a range of weight‐based dosing increments in patients with significant eGFR thresholds.

Finally, for longitudinal mixed‐effects modeling, we included patients in the Wisconsin registry with recurrent visits and eGFR ≤60 mL/min/1.73 m^2^ (CKD stage ≥3) and who had data on eGFR and HCQ blood levels at each visit (n = 32 unique patients, median visit frequency = 2). Using these longitudinal (recurrent visit) data, we performed a linear mixed‐effects model analysis with random intercepts to estimate the change in HCQ blood levels per unit eGFR decline relative to HCQ dose category (>5 vs ≤5 mg/kg/day). These models were adjusted for age, sex, and weight‐based HCQ dose.

## RESULTS

### Baseline characteristics of population

Among 2,010 total patients, 168 patients with very low HCQ levels <200 ng/mL were excluded, and a total of 1,842 patients were included in the main analysis (238 patients from the Wisconsin registry [United States], 1,002 patients from three different published studies centralized in France, and 602 patients from the SLICC cohort). Forty‐six percent of the study population was from the multicenter international SLICC cohort and the US cohort. The mean ± SD age was 39 ± 14, 90.4% were female, 56% were White, 29% were Black, 10% were from other ethnic groups, the mean ± SD weight was 68 ± 17 kg, the mean ± SD HCQ dose was 349 ± 88 mg/day, and 44% of patients were taking a ≤5‐mg/kg/day HCQ dose. On the day of HCQ blood level measurement (T0), 559 patients had active SLE (SLEDAI‐2K score ≥6). Baseline characteristics are shown in Table 1 (all cohorts) and Supplementary Table 1 (by data sets/cohorts).

**Table 1:** Baseline characteristics of the participants*

	Value
Baseline characteristics (baseline visit = T0)	
Participants included in analysis, N	1,842^a^
Age in years, mean ± SD	39 ± 14
Sex, n (%)	
Female	1,665 (90.4)
Male	177 (8.6)
Race and ethnicity, n (%)	
Asian	54 (3)
Black	541 (29)
Hispanic	31 (2)
Other racial and ethnic groups	189 (10)
White	1,027 (56)
Weight in kg, mean ± SD	68 ± 17
eGFR in mL/min/1.73 m^2^, mean ± SD	102 ± 33
eGFR ≥60 mL/min/1.73 m^2^, n (%)	1,738 (94.4)
eGFR ≥45 and <60 mL/min/1.73 m^2^, n (%)	55 (3.0)
eGFR <45 mL/min/1.73 m^2^, n (%)	49 (2.6)
HCQ dose in mg/day, mean ± SD	349 ± 88
HCQ dose ≤5 mg/kg/day, n (%)	781 (42)
HCQ dose >5 mg/kg/day, n (%)	1,061 (58)
Cumulative HCQ dose in g, mean ± SD	2,068 ± 1,053
Total HCQ duration in years, mean ± SD	16.0 ± 6.8
HCQ blood levels at T0 in ng/mL, mean ± SD^b^	916 ± 424
Outcome variables	
SLEDAI at baseline visit (T0), mean ± SD	4.1 ± 4.6
Active lupus (SLEDAI score ≥6) at baseline visit (T0), n/N (%)	559/1,842 (30)
Systemic^c^ HCQ‐related toxicity^d^ at T_lastvisit_, n/N (%)	91/1,842 (4.9)

* eGFR was calculated using the race‐neutral Chronic Kidney Disease Epidemiology Collaboration 2021 equation. eGFR, glomerular filtration rate; HCQ, hydroxychloroquine; SLEDAI, Systemic Lupus Erythematosus Disease Activity Index; SLICC, Systemic Lupus International Collaborating Clinics; T0, at the time of enrollment; T_lastvisit_, at the time of the last follow‐up visit.

^a^ One hundred sixty‐eight patients with very low HCQ whole blood levels <200 ng/mL (shown to be associated with severe nonadherence) were excluded from analysis.

^b^ HCQ blood levels were extrapolated from serum levels for the SLICC cohort using a conversion factor of 0.53.

^c^ Includes all systemic toxicity, 85% retinopathy, 9% cardiomyopathy, and 6% skin or muscle toxicity.

^d^ One cohort (SLICC) only reported retinopathy as HCQ‐related systemic toxicity, one study (multicentric international, n = 305 patients) did not report long‐term toxicity data, and a single condition imputation analysis was used to impute toxicity for this cohort using the median HCQ toxicity incidence rate (4.7%).

### HCQ blood levels and systemic toxicity

**Figure 1:**
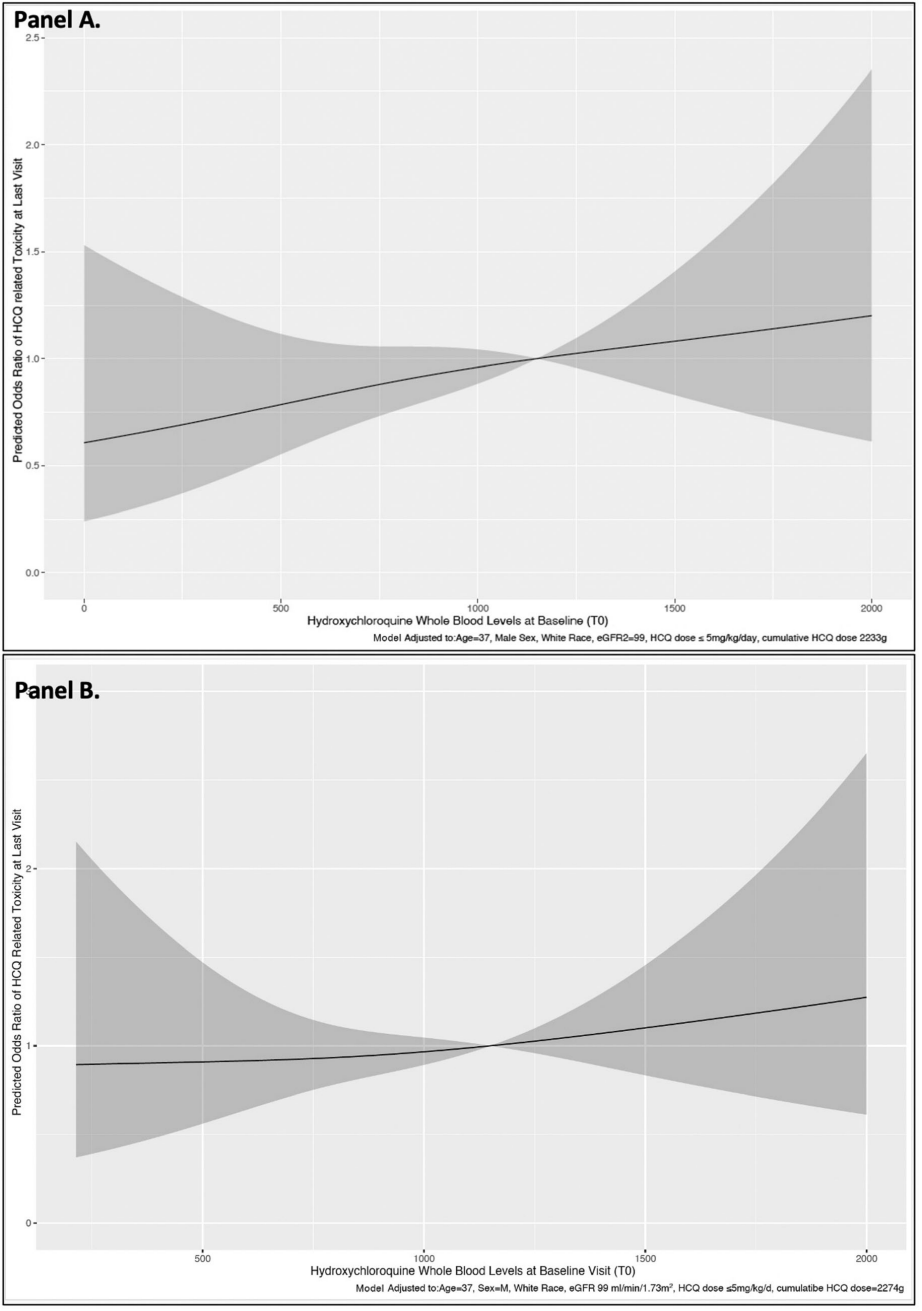
Adjusted restricted cubic splines to show associations between HCQ whole blood levels at baseline and HCQ‐related toxicity over time using data from (A) 1,842 patients (not including 168 patients with very low HCQ blood levels <200 ng/mL) and (B) all patients (N = 2,010). eGFR, estimated glomerular filtration rate; HCQ, hydroxychloroquine.

The overall HCQ‐related toxicity rate was 4.9% (1% in Wisconsin cohort, 6.2% in data from three previously published studies [France and international], 4.7% in the SLICC cohort). After excluding data from 305 patients without toxicity data, the HCQ‐related toxicity rate was 5.1%. Given similar results, we show the results from the single conditional imputation analysis. The overall HCQ retinopathy was 4.2%. Using the cut point analysis, we found that levels ≥1,150 ng/mL had a ≥93% NPV of identifying toxicity, indicating a potential optimal cut point or upper threshold for HCQ blood levels that could be associated with higher toxicity. Next, the estimated OR of toxicity using 1,150 ng/mL as a reference point revealed a linear increase in estimated OR of toxicity for levels ≥1,150 ng/mL (Figure 1A). Finally, comparing levels informed by the aforementioned analysis, levels ≥1,150 ng/mL were associated with 2.1‐fold higher odds of toxicity compared to levels of 750 to <1,150 ng/mL (Table 2), even after adjusting for covariables. These analyses underscored 1,150 ng/mL as an optimal upper threshold for HCQ blood levels linked with toxicity. Similar associations were noted when all patients were included (N = 2,010) in sensitivity analyses (Supplementary Table 2A and Figure 1B). Similar results were noted when serum levels in the SLICC cohort and blood levels from the Wisconsin registry and three published studies (France and international) were analyzed separately (Supplementary Figures 1A and 2A). Finally, even after excluding data from one study that did not have HCQ toxicity information, similar odds of toxicity 1.9‐fold (95% confidence interval [CI] 1.2–3.1) were noted with HCQ whole blood levels ≥1,150 ng/mL. Likewise, even after including only HCQ retinopathy, 1.3‐fold higher odds of HCQ retinopathy were noted with HCQ whole blood levels ≥1,150 ng/mL (adjusted OR = 1.3, 95% CI 1.2–3.3, *P* = 0.007).

**Table 2:** Multivariable logistic regression analysis showing factors at baseline visit (T0) associated with HCQ toxicity over time using data from different populations (n = 1,842)*

Variables	Adjusted OR^a^ (95% CI)	*P* value
**Age per 10‐y increase**	**1.02 (1.01–1.05)**	**0.02**
Female	0.80 (0.42–1.67)	0.53
White race	Ref	–
Black race	0.97 (0.59–1.57)	0.90
Asian race	0.90 (0.14–3.13)	0.89
Other race or ethnicity	1.67 (0.73–3.50)	0.19
Hispanic ethnicity	NA^b^	0.98
Weight‐based HCQ dose, >5 mg/kg/day	0.82 (0.47–1.43)	0.48
**Cumulative HCQ dose per 1,000‐g increase**	**1.74 (1.34–2.30)**	**<0.0001**
eGFR per 10‐mL/min/1.73 m^2^ increase	0.93 (0.85–1.01)	0.10
Therapeutic HCQ blood levels 750 to <1,150 ng/mL	Ref^c^	–
Subtherapeutic HCQ blood levels <750 ng/mL	1.40 (0.80–2.51)	0.24
**Supratherapeutic HCQ blood levels ≥1,150 ng/mL**	**2.09 (1.22–3.67)**	**0.01**

* One hundred sixty‐eight patients with very low HCQ whole blood levels <200 ng/mL (shown to be associated with severe nonadherence) were excluded from analysis. Statistically significant *P* values (<0.05) are shown in bold. CI, confidence interval; eGFR, estimated glomerular filtration rate; HCQ, hydroxychloroquine; NA, not applicable; OR, odds ratio.

^a^ The model was adjusted for age (continuous, T0), sex (patient reported, T0), race or ethnicity, weight‐based HCQ dose (>5 vs ≤5 [reference group] mg/kg/day at T0), eGFR (continuous at T0), HCQ whole blood level categories (750 to <1,150 [reference group] vs <750 vs ≥1,150 ng/mL at T0), and cumulative HCQ dose (continuous and calculated between baseline visit and last visit or day of HCQ toxicity [T_last visit_]).

^b^ Unreliable estimates due to small sample size.

^c^ The therapeutic range for HCQ blood levels of 750 to <1,150 ng/mL was used as a reference group to demonstrate the ceiling or saturation effect in clinical response with levels >1,150 ng/mL and to demonstrate higher odds of active systemic lupus erythematosus with levels below the therapeutic range.

### Supratherapeutic HCQ whole blood levels

**Figure 2:**
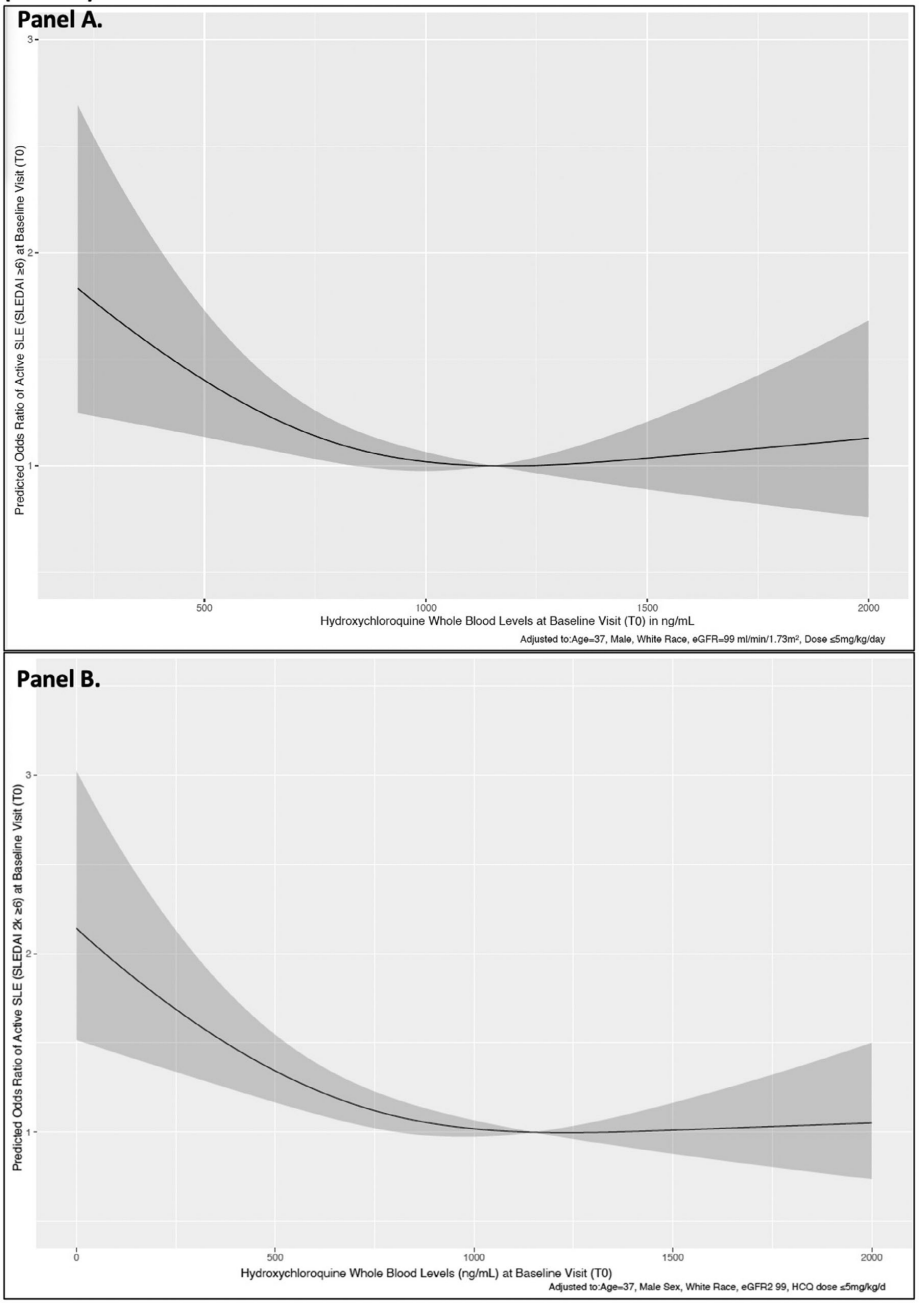
Adjusted restricted cubic splines to show associations between HCQ whole blood levels at baseline visit (T0) and active SLE (SLEDAI score ≥6) at baseline visit (T0) using data from (A) 1,842 patients (not including 168 patients with very low HCQ blood levels <200 ng/mL) and (B) all patients (N = 2,010). eGFR, estimated glomerular filtration rate; HCQ, hydroxychloroquine; SLE, systemic lupus erythematosus; SLEDAI, Systemic Lupus Erythematosus Disease Activity Index.

We then tested if this HCQ blood level threshold linked with higher toxicity was associated with any additional benefits in reducing odds of active SLE, defined as an SLEDAI‐2K score ≥6. Using cut point analysis, levels 1,150 ng/mL had an NPV of 96% for active lupus (SLEDAI‐2K score ≥6). Next, we completed a restricted cubic spline analysis using 1,150 ng/mL as the reference point. We noted only a slight change in estimated active lupus (SLEDAI‐2K scores ≥6) odds with levels above 1,150 ng/mL (Figure 2A). These findings highlighted a ceiling effect at 1,150 ng/mL. Thus, in our multivariable analysis, we used the HCQ levels categories <750 versus 750 to <1,150 versus ≥1,150 ng/mL, informed by the aforementioned findings and published literature, including a global meta‐analysis. In logistic regression analysis adjusted for variables that could potentially lead to active lupus and using 750 to <1,150 ng/mL as the reference category, no significant reduction in odds of active lupus was noted with levels ≥1,150 ng/mL (Table 3), whereas subtherapeutic levels of <750 ng/mL were associated with 1.36‐fold higher odds of active lupus (Table 3). This finding suggests that levels beyond this threshold (≥1,150 ng/mL) were indeed supratherapeutic and potentially associated with higher risk of toxicity. Finally, an HCQ dose >5 mg/kg/day was associated with 0.54‐fold lower odds of active lupus (Table 3). Similar associations were noted when all patients were included (N = 2,010) in sensitivity analyses (Supplementary Table 2B and Figure 2B) and when serum levels in the SLICC cohort and blood levels from the Wisconsin registry and three published studies (France and international) were analyzed separately (Supplementary Figures 1B and 2B).

**Table 3:** Multivariable logistic regression analysis showing factors at baseline visit (T0) associated with active SLE (SLEDAI‐2K score ≥6) at baseline visit (T0) using data from different populations (n = 1,842)*

Variables	Adjusted OR^a^ (95% CI)	*P* value
**Age per 10‐y increase**	**0.99 (0.98–1.00)**	**0.02**
Female	1.44 (1.00–2.09)	0.05
White race	Ref	–
Black race	0.95 (0.74–1.21)	0.68
**Asian race**	**4.12 (2.32–7.55)**	**<0.0001**
**Other race or ethnicity**	**2.17 (1.56–3.03)**	**<0.0001**
Hispanic ethnicity	1.87 (0.88–3.89)	0.10
**Weight‐based HCQ dose, >5 mg/kg/day**	**0.54 (0.44–0.68)**	**<0.0001**
eGFR per 10‐mL/min/1.73 m^2^ increase	1.01 (0.97–1.05)	0.72
Therapeutic HCQ blood levels 750 to <1,150 ng/mL	Ref^b^	–
**Subtherapeutic HCQ blood levels <750 ng/mL**	**1.33 (1.05–1.70)**	**0.02**
Supratherapeutic HCQ blood levels ≥1,150 ng/mL	0.94 (0.71–1.24)	0.67

* One hundred sixty‐eight patients with very low HCQ whole blood levels <200 ng/mL (shown to be associated with severe nonadherence) were excluded from analysis. Statistically significant *P* values (<0.05) are shown in bold. CI, confidence interval; eGFR, estimated glomerular filtration rate; HCQ, hydroxychloroquine; OR, odds ratio; SLE, systemic lupus erythematosus; SLEDAI‐2K, Systemic Lupus Erythematosus Disease Activity Index 2000.

^a^ The model was adjusted for covariables at baseline visit (T0), including age (continuous, T0), sex (patient reported, T0), race or ethnicity (T0), weight‐based HCQ dose (>5 vs ≤5 [reference group] mg/kg/day at T0), eGFR (continuous at T0), and HCQ whole blood level categories (750 to <1,150 [reference group] vs <750 vs ≥1,150 ng/mL at T0). SLEDAI‐2K scores at baseline visit (T0) were used as the outcome and categorized as active SLE (SLEDAI‐2K score ≥6).

^b^ The therapeutic range for HCQ blood levels of 750 to <1,150 ng/mL was used as a reference group to demonstrate the ceiling or saturation effect in clinical response with levels >1,150 ng/mL and to demonstrate higher odds of active SLE with levels below the therapeutic range.

### Associations between weight‐based HCQ dosing, kidney function thresholds, and supratherapeutic HCQ blood levels

**Figure 3:**
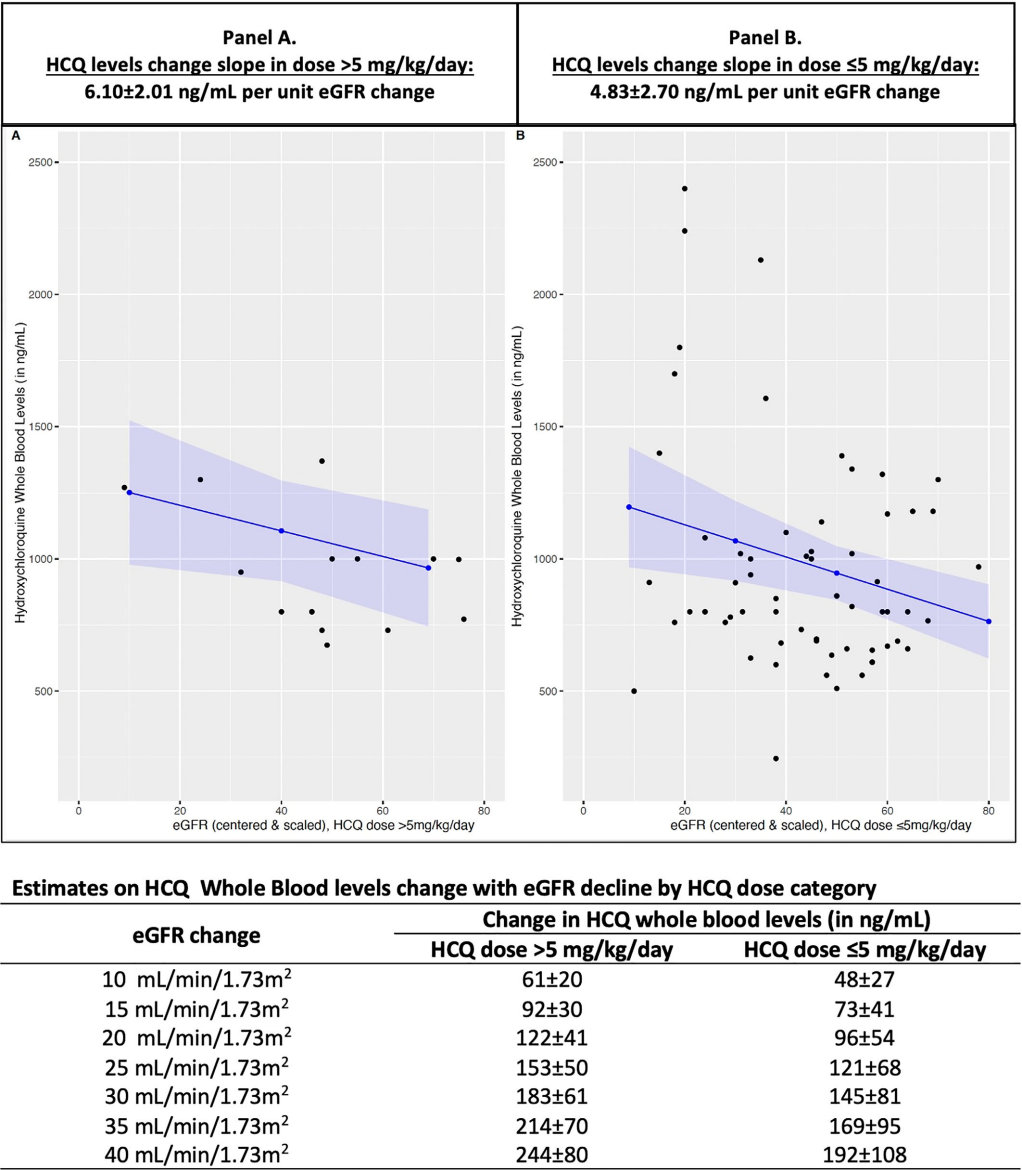
Longitudinal modeling showing change in HCQ whole blood levels with kidney function decline over time in patients with CKD (32 patients with CKD stage ≥3 had two or more follow‐up visits) by HCQ daily dose category: (A) >5 mg/kg/day versus (B) ≤5 mg/kg/day. CKD, chronic kidney disease; eGFR, estimated glomerular filtration rate; HCQ, hydroxychloroquine.

We noted a significant number of patients (n = 142 [18%]) had supratherapeutic and potentially toxic levels ≥1,150 ng/mL despite weight‐based dosing (Supplementary Figure 3A), whereas 37% had supratherapeutic levels with doses >5 mg/kg/day (Supplementary Figure 3B). Next, adjusted logistic regression analyses noted an eGFR threshold of <60 mL/min/1.73 m^2^ was associated with 2.3‐fold higher odds of having supratherapeutic HCQ blood levels (Supplementary Table 3). In the CKD Stage 3a (eGFR 45–59 mL/min/1.73 m^2^) and CKD Stage 3b or above (eGFR <45 mL/min/1.73 m^2^) subgroups, a positive association was noted between weight‐based HCQ daily dose (mg/kg/day) and predicted HCQ whole blood levels (ng/mL), with an estimated mean ± SD slope of 136 ± 59 ng/mL and 166 ± 59 ng/mL per 1‐mg/kg/day increase, respectively (Supplementary Figure 4). In patients with CKD Stage 3a and CKD Stage 3b or above, even with weight‐based dosing of 5 mg/kg/day, the predicted HCQ blood levels were near the supratherapeutic (toxic) threshold. A slight increase in the dose or decline in eGFR would tip levels over to the supratherapeutic threshold, risking toxicity over time in patients with CKD Stage 3 or above (Supplementary Figure 4). This was validated in our adjusted linear longitudinal mixed‐effects model, which showed a steeper slope of change in HCQ blood levels relative to eGFR decline in patients with an eGFR <60 mL/min/1.73 m^2^ and taking an HCQ dose >5 mg/kg/day (mean ± SD 6.11 ± 2.04 ng/mL) versus those taking a dose of ≤5 mg/kg/day (mean ± SD 4.82 ± 2.72 ng/mL) (Figure 3). Per these slope estimates, significant changes in eGFR, such as a 20‐unit decrease, would result in a 122‐ng/mL (95% CI 82–162 ng/mL) and 96‐ng/mL (95% CI 42–150 ng/mL) increase in HCQ blood levels with doses of >5 and ≤5 mg/kg/day, respectively.

## DISCUSSION

In this study leveraging cross‐sectional data from diverse SLE cohorts, HCQ whole blood levels ≥1,150 ng/mL at T0 (baseline) were associated with a 2.1‐fold higher risk of systemic HCQ‐related toxicity. HCQ‐related systemic toxicity was infrequent, and only 4.9% of the population experienced toxicity; therefore, we tested the therapeutic effect of having levels ≥1,150 ng/mL. We noted that levels ≥1,150 ng/mL did not significantly reduce the odds of active SLE, indicating a ceiling effect in clinical response with levels ≥1,150 ng/mL and qualifying levels ≥1,150 ng/mL as supratherapeutic levels. Finally, our findings highlight that CKD stage ≥3 was associated with 2.3‐fold higher odds of having supratherapeutic levels (≥1,150 ng/mL), and not adjusting HCQ dose in this group could significantly increase levels by 122 ng/mL, with eGFR decline of 20 units. Together these findings establish the clinical significance of HCQ blood level monitoring in not only assessing for severe nonadherence but also guiding clinicians with optimal HCQ use to balance treatment efficacy and safety. Finally, a 2020 single‐center prospective cohort study led by Dr Petri^19^ highlighted that HCQ whole blood levels ≥1,200 ng/mL were associated with higher risk of retinopathy. Those findings are consistent with this study's findings. These findings suggest that careful HCQ dose adjustments via HCQ blood level monitoring, particularly in high‐risk groups (eg, CKD stage ≥3), could be beneficial in personalizing HCQ dosing. Additional multicenter longitudinal studies are needed in the future to establish a roadmap for dose adjustments based on individual patient risk factors.

Following the first study published in 2006 showing that low HCQ levels predict disease exacerbations in patients with SLE,^33^ studies from other lupus cohorts^11^, ^13^, ^25^, ^34^ and a global meta‐analysis^9^ confirmed 750 or 1,000 ng/mL as a lower threshold for the reference range for HCQ blood levels associated with better efficacy. In 2020, evidence on the upper threshold of the reference range for HCQ blood level monitoring was reported, and levels >1,183 ng/mL were associated with higher retinopathy risk (incidence of retinopathy = 4.3%).^19^ Consistent with prior findings, the current study highlights the reliability of the upper threshold (≥1,150 ng/mL) of the reference range for HCQ blood levels by establishing associations with higher toxicity risk without additional clinical benefits across global SLE populations (N = 2,010). Although a portion of the data was derived from French cohorts, over 46% of the study population was drawn from the multicenter international SLICC cohort and a US‐based registry, enhancing the generalizability of our findings. The demographic characteristics of the cohort, including a mean ± SD age of 39 ± 14 years, 90.4% female representation, and racial and ethnic diversity with 56% White, 29% Black, and 10% from other racial or ethnic groups, are consistent with the known epidemiology of SLE. Additionally, the mean ± SD body weight (68 ± 17 kg) and HCQ dosing patterns reflect real‐world clinical practice, supporting the applicability of our findings to broader SLE populations.

Routine HCQ whole blood level monitoring bypasses clinical variables (eg, absorption, clearance) that can drive interindividual variability in drug levels, is easier to collect, and guides clinicians in adjusting HCQ doses when levels are supratherapeutic, particularly in patients at higher risk of HCQ toxicity, such as those with CKD.^30^ This study provides important data highlighting that 18% of patients had supratherapeutic and potentially toxic levels despite taking ≤5 mg/kg/day of HCQ (44% of the total population). This finding underscores a role of HCQ blood level monitoring even in patients with weight‐based dosing. This study sheds new evidence on thresholds of kidney function (eGFR <60 mL/min/1.73 m^2^) associated with a 2.3‐fold higher risk of supratherapeutic (or toxic) HCQ blood levels. This threshold highlights the need to adjust HCQ doses or perform closer monitoring in patients with low kidney function (eGFR <60 mL/min/1.73 m^2^).

Additionally, our study highlights a dose‐dependent relationship in patients with impaired renal function (CKD Stage 3a or above) and may have implications for individualized dosing strategies in the CKD subgroup. Although data on how HCQ dose should be reduced in patients with CKD to balance efficacy versus toxicity are lacking, our study delivers preliminary data on changes in HCQ blood levels with eGFR decline relative to weight‐based HCQ dose over time. In patients with CKD stage ≥3 taking HCQ doses >5 mg/kg/day, a clinically significant increase in HCQ blood levels by 122 ng/mL was noted with an eGFR decline of 20 units. Despite HCQ dosing (>5 or ≤5 mg/kg/day), variations in HCQ blood levels with eGFR decline of 20 units were above the diurnal variation threshold of <80 ng/mL. Moreover, we noted that patients with eGFR <60 mL/min/1.73 m^2^ had higher chances of having supratherapeutic (toxic) levels even if they were receiving weight‐based dosing of ≤5 mg/kg/day, as shown in Figure 3. Thus, close monitoring of HCQ blood levels to guide HCQ dose adjustments could prevent toxicity^29^ and maximize efficacy in patients with SLE and CKD stage ≥3. Our preliminary findings, along with recent interpretation of HCQ blood levels offered by Balevic et al, provide essential data to design a future clinical trial leveraging HCQ blood level monitoring to inform dose or formulation changes in patients with SLE and CKD.^14^ However, given the variability in HCQ blood levels, especially in case of variation in adherence to treatment or even eGFR fluctuations,^14^ a decision to adjust HCQ dose, particularly when the dose is weight based, should not be made based on a single HCQ blood level measurement. We recommend repeat measurement of HCQ levels to obtain a better estimate on the median levels and inform clinical decision‐making.

The feasibility of HCQ blood level monitoring in the target population is an important consideration. In our view, incorporating HCQ blood level monitoring into clinical care is feasible and is already being routinely implemented in France, other parts of Europe, and some US centers. Supporting this, qualitative studies reported that patients are generally receptive to drug level monitoring, particularly when clinicians initiate open, empathetic conversations during visits.^35^, ^36^, ^37^ Moreover, HCQ level testing is covered by the public health system in France (cost <€30) and by most insurance providers in the United States (copay cost $0–200) and has an established CPT code (80220) in the United States, which supports its practical implementation. Furthermore, drug level monitoring is already a standard practice in other specialties, such as gastroenterology, nephrology, and transplantation medicine, in which patient acceptance has been high. It is also close to the monitoring of international normalized ratio in patients taking warfarin. Finally, HCQ level testing should guide nonjudgmental communication and support physicians and patients in optimizing HCQ dosing.^38^ Indeed, in addition to giving important information on adherence to treatment, HCQ level monitoring can offer clinicians the opportunity to optimize HCQ dosing based on each patient's factors, such as CKD and absorption, which are otherwise not accounted for when routinely dosing HCQ. This approach, already used in France for more than 20 years, could shift from traditional “one‐size‐fits‐all” dosing toward a more precise, personalized approach to HCQ management in SLE. Two US longitudinal, prospective mixed‐methods studies are currently under way (SHIELD and EMS‐HCQ), and a French study is currently being designed to compare both strategies (weight‐based HCQ dosing vs personalized HCQ dosing per HCQ blood levels). These ongoing studies will provide valuable insights on HCQ blood level monitoring frequency, cost‐effectiveness, and patient–clinician engagement to develop best practices. Additionally, a shared decision‐making tool that guides clinicians and patients in decisions to continue HCQ dose with or without changes could be a vital resource for clinical use and should be a research priority.

Despite several strengths of this study, including a large study with diverse populations and appropriate testing of HCQ levels, study limitations include the retrospective design and the inability to control for all confounders affecting outcomes, such as other immunosuppressive medications and steroid dose. Additionally, whole blood levels in the primary population were assayed in each local laboratory as part of routine practice. We cannot demonstrate the interchangeability of all bioanalytical methods due to the lack of an external quality assessment system between laboratories, which is a limitation of our analysis. Besides, HCQ serum levels in the SLICC cohort were measured retrospectively, and whole blood levels were extrapolated from serum levels, which can represent a bias in our analysis because there could be stability issues of HCQ serum levels over time and potential variability introduced by individual hematocrit differences, and the implications for interpreting our findings. Third, the data on the time to HCQ toxicity and HCQ levels at the time of HCQ toxicity were not available. Given the goal of HCQ levels is to determine the chance of future toxicity, these findings remain significant. Moreover, the supratherapeutic effect of the upper threshold for HCQ levels (≥1,150 ng/mL) was tested using HCQ levels and SLEDAI‐2K measurements done on the same day (T0). This bolsters the fact that the upper threshold is supratherapeutic and potentially toxic. However, given that our study shows that HCQ levels could vary with significant kidney function decline and given that our models’ testing associations with HCQ toxicity assumed that HCQ levels remain stable over time, we need to test the upper threshold in longitudinal studies, particularly in patients with CKD stage ≥3. Additionally, longitudinal studies should test if a single HCQ blood level measurement versus an average of two or more HCQ blood level measurements should be used to guide HCQ dose adjustments. Fourth, one cohort did not report on long‐term eye toxicity; therefore, a single condition imputation was performed to avoid sample size reduction. Results from the single condition imputation analysis and sensitivity analysis excluding data from this cohort were similar. Additionally, our study might underestimate the overall HCQ‐related side effects rate given that data on preclinical HCQ toxicity (abnormal eye examination) might not have been abstracted. Finally, only a few patients with CKD stage ≥3 had recurrent visit data (n = 32). Thus, a prospective study is needed to test the efficacy of HCQ blood level monitoring in guiding safe and optimal HCQ use in patients with CKD and SLE, and similar analyses in patients with hepatic dysfunction and gastric bypass are needed.

In conclusion, this study is the first to define a potential therapeutic reference range for HCQ whole blood levels (750–1,150 ng/mL) in SLE, using data from diverse multinational cohorts. It also identifies key patient‐specific factors, particularly CKD stage ≥3, that significantly increase the risk of supratherapeutic levels, despite weight‐based dosing. By providing quantitative estimates of how HCQ levels change with dose and kidney function decline, our findings offer clinicians a practical framework for safer and more individualized, data‐driven dosing decisions. Although our data are cross‐sectional, they still lay a strong foundation for future longitudinal studies to evaluate the clinical effectiveness of routine HCQ blood level monitoring. Ultimately, this work supports a shift from traditional “one‐size‐fits‐all” dosing toward a more balanced, precise, and personalized approach to dose HCQ in SLE, potentially bypassing the current issues with weight‐based dosing.

## AUTHOR CONTRIBUTIONS

All authors contributed to at least one of the following manuscript preparation roles: conceptualization AND/OR methodology, software, investigation, formal analysis, data curation, visualization, and validation AND drafting or reviewing/editing the final draft. As corresponding authors, Drs Garg and Costedoat‐Chalumeau confirm that all authors have provided the final approval of the version to be published and take responsibility for the affirmations regarding article submission (eg, not under consideration by another journal), the integrity of the data presented, and the statements regarding compliance with institutional review board/Declaration of Helsinki requirements.

## Supporting information


**Disclosure Form**:

**Data S1** Supporting Information.
