# Supplementary material for: Defining Optimally Safe and Effective Blood Levels of Hydroxychloroquine in Lupus: An Important Step Toward Precision Drug Monitoring
Source: Arthritis Rheumatol. 2026 Feb 17;78(5):1102–13. doi: 10.1002/art.70010 (PMC13129619; doi:10.1002/art.70010)
Supplement: Supplementary file 2 — Data S1 Supporting Information. [file ART-78-1102-s002.docx]

**Supplementary Table 1. HCQ dose, cumulative HCQ exposure, and HCQ levels by datasets^1^**

| **Variables** | **Wisconsin** | **France & International** | **SLICC cohort** |
| --- | --- | --- | --- |
| Total Number | 238 | 1002 | 602 |
| Age in years (mean±SD) | 47±17 | 38±12 | 37±14 |
| White race (%) | 70% | 52% | 55% |
| Female (%) | 90% | 92% | 88% |
| HCQ dose in mpk/d (mean±SD) | 4.3±1.1 | 5.7±1.9 | 5.2±1.6 |
| Cumulative HCQ dose in g (mean±SD) | 893±688 | 2734±737 | 1423±776 |
| HCQ levels in ng/mL (mean±SD) | 925±397 | 956±432 | 845±410 |
| HCQ related toxicity (%) | 1% | 6.2% | 4.7% |
| HCQ retinopathy (%) | 1% | 5.3% | 4.7% |

*Abbreviations: yrs=years; mpk/d=mg per kg/day; HCQ=Hydroxychloroquine*

*168 patients with very low HCQ whole blood levels <200 ng/mL that have been associated with severe nonadherence were excluded from analysis.*

| **Supplementary Table 2A. Multivariable logistic regression analysis showing factors at baseline visit (T0) associated with HCQ toxicity over time including all patients (n=2010)** | | |
| --- | --- | --- |
| Variables | Adjusted OR^A^ (95% CIs) | p-value |
| **Age per 10 years increase** | **1.02 (1.00-1.04)** | **0.02** |
| Female | 0.84 (0.44-1.73) | 0.60 |
| White Race | Ref | - |
| Black Race | 1.02 (0.63-1.63) | 0.94 |
| Asian Race | 0.86 (0.14-2.97) | 0.84 |
| Other Race or Ethnicity | 1.60 (0.70-3.33) | 0.23 |
| Hispanic Ethnicity | NA^B^ | 0.98 |
| Weight based HCQ dose, >5mg/kg/day | 0.76 (0.44-1.30) | 0.31 |
| **Cumulative HCQ dose per 1000 g increase** | **1.72 (1.33-2.24)** | **<0.0001** |
| eGFR per 10 mL/min/1.73m^2^ increase | 0.93 (0.86-1.02) | 0.11 |
| Therapeutic HCQ Blood levels 750-<1150 ng/mL | Ref^C^ | - |
| Very low HCQ Blood levels <200 ng/mL | 0.66 (0.15-1.98) | 0.52 |
| Subtherapeutic HCQ Blood levels 200-749 ng/mL | 1.39 (0.75-2.48) | 0.26 |
| **Supratherapeutic** **HCQ Blood levels ≥1150 ng/mL** | **2.11 (1.23-3.70)** | **0.01** |

| **Supplementary Table 2B. Multivariable logistic regression analysis showing factors at baseline visit (T0) associated with active SLE (SLEDAI ≥6) at baseline visit (T0) including all patients (n=2010)** | | |
| --- | --- | --- |
| Variables | Adjusted OR^D^ (95% CIs) | p-value |
| **Age per 10 years increase** | 0.99(0.98-1.00) | **0.001** |
| Female | 1.33 (0.95-1.89) | 0.10 |
| White Race | Ref | - |
| Black Race | 0.99 (0.79-1.24) | 0.93 |
| **Asian Race** | 3.02 (1.77-5.23) | **<0.0001** |
| **Other Race or Ethnicity** | 2.00 (1.46-2.74) | **<0.0001** |
| Hispanic Ethnicity | 1.53 (0.78-2.95) | 0.21 |
| **Weight based HCQ dose, >5mg/kg/day** | **0.57 (0.46-0.70)** | **<0.0001** |
| eGFR per 10 mL/min/1.73m^2^ increase | 1.01 (0.98-1.05) | 0.54 |
| Therapeutic HCQ Blood levels 750-<1150 ng/mL | Ref^C^ | - |
| **Very low HCQ Blood levels <200 ng/mL** | **1.98 (1.38-2.85)** | **0.0002** |
| **Subtherapeutic HCQ Blood levels <750 ng/mL** | **1.35 (1.06-1.72)** | **0.02** |
| Supratherapeutic HCQ Blood levels ≥1150 ng/mL | 0.95 (0.72-1.25) | 0.69 |

***Footnotes A-D:***

***A:*** *The model was adjusted for age (continuous, T0), sex (patient-reported, T0), race or ethnicity, weight-based HCQ dose (>5 vs. ≤5 (reference group) mg/kg/day at T0), eGFR (continuous at T0), HCQ whole blood level categories (750-<1150 (reference group) vs. <200 vs. 200-749 vs. ≥1150 ng/mL at T0), cumulative HCQ dose (continuous and calculated between baseline visit and last visit or day of HCQ toxicity, Tlast visit).*

***B:*** *Unreliable estimates due to small sample size.*

***C:*** *Therapeutic range for HCQ blood levels 750-<1150* *ng/mL was used as a reference group to demonstrate the ceiling or saturation effect in active SLE (clinical response) with levels ≥1150 ng/mL and demonstrate higher odds of active SLE with levels below therapeutic range*

***D:*** *The model in Table 2B was adjusted for covariables at baseline visit (T0) including age (continuous, T0), sex (patient-reported, T0), race or ethnicity (T0), weight-based HCQ dose (>5 vs. ≤5 (reference group) mg/kg/day at T0), eGFR (continuous at T0), and HCQ whole blood level categories (750-1149 (reference group) vs. <750 vs. ≥1150 ng/mL at T0). SLEDAI-2K scores at baseline visit (T0) were used as the outcome and categorized as active SLE (SLEDAI-2K ≥6).*

*P-values (<0.05) shown in bold. HCQ=Hydroxychloroquine; eGFR=estimated glomerular filtration rate.*

| **Supplementary Table 3. Multivariable logistic regression analysis showing factors at baseline visit (T0) associated with supratherapeutic (or toxic) HCQ whole blood levels, ≥1150 ng/mL, at baseline visit (T0) across 1842^A^ patients** | | |
| --- | --- | --- |
| Variables | Adjusted OR^B^ (95% CIs) | p-value |
| **Age per 10 years increase** | 1.01 (1.00-1.02) | 0.10 |
| Female | 1.37 (0.93-2.08) | 0.13 |
| White Race | Ref | - |
| **Black Race** | **1.29 (1.01-1.63)** | **0.04** |
| Asian Race | 1.28 (0.65-2.39) | 0.46 |
| Other Race or Ethnicity | 0.88 (0.60-1.29) | 0.52 |
| Hispanic Ethnicity | 1.34 (0.55-2.98) | 0.49 |
| **Weight based HCQ dose, >5mg/kg/day** | **2.35 (1.79-3.10)** | **<0.0001** |
| Weight in Kg | 1.00 (0.99-1.01) | 0.82 |
| eGFR category ≥60 ml/min/1.73m^2^ | Ref^C^ | - |
| **eGFR category 45-59 ml/min/1.73m^2^** | **2.11 (1.14-3.85)** | **0.02** |
| **eGFR category <45 ml/min/1.73m^2^** | **2.45 (1.31-4.47)** | **0.004** |

***Footnotes A-C:***

***A:*** *168 patients with very low HCQ whole blood levels <200 ng/mL (shown to be associated with severe nonadherence) were excluded from analysis*

***B:*** *This model was adjusted for age (continuous), sex (patient-reported), weight-based HCQ dose (>5 vs. ≤5 (reference group) mg/kg/day), eGFR categorized as ≥60 (reference category) vs. 45-59 vs. <45 ml/min/1.73m2.*

***C:*** *eGFR categorized as ≥60 (reference category)*

*Statistically significant p-values (<0.05) shown in bold font.*

*Abbreviations: HCQ=Hydroxychloroquine; eGFR=estimated glomerular filtration rate.*

**Supplementary Figure 1. Adjusted restricted cubic splines to show associations between HCQ whole blood levels and toxicity (Panel A), and active SLE (SLEDAI ≥6) (Panel B), across 1240 patients (including data from Wisconsin registry and three previous studies centralized in France).**

**Panel A.**

*
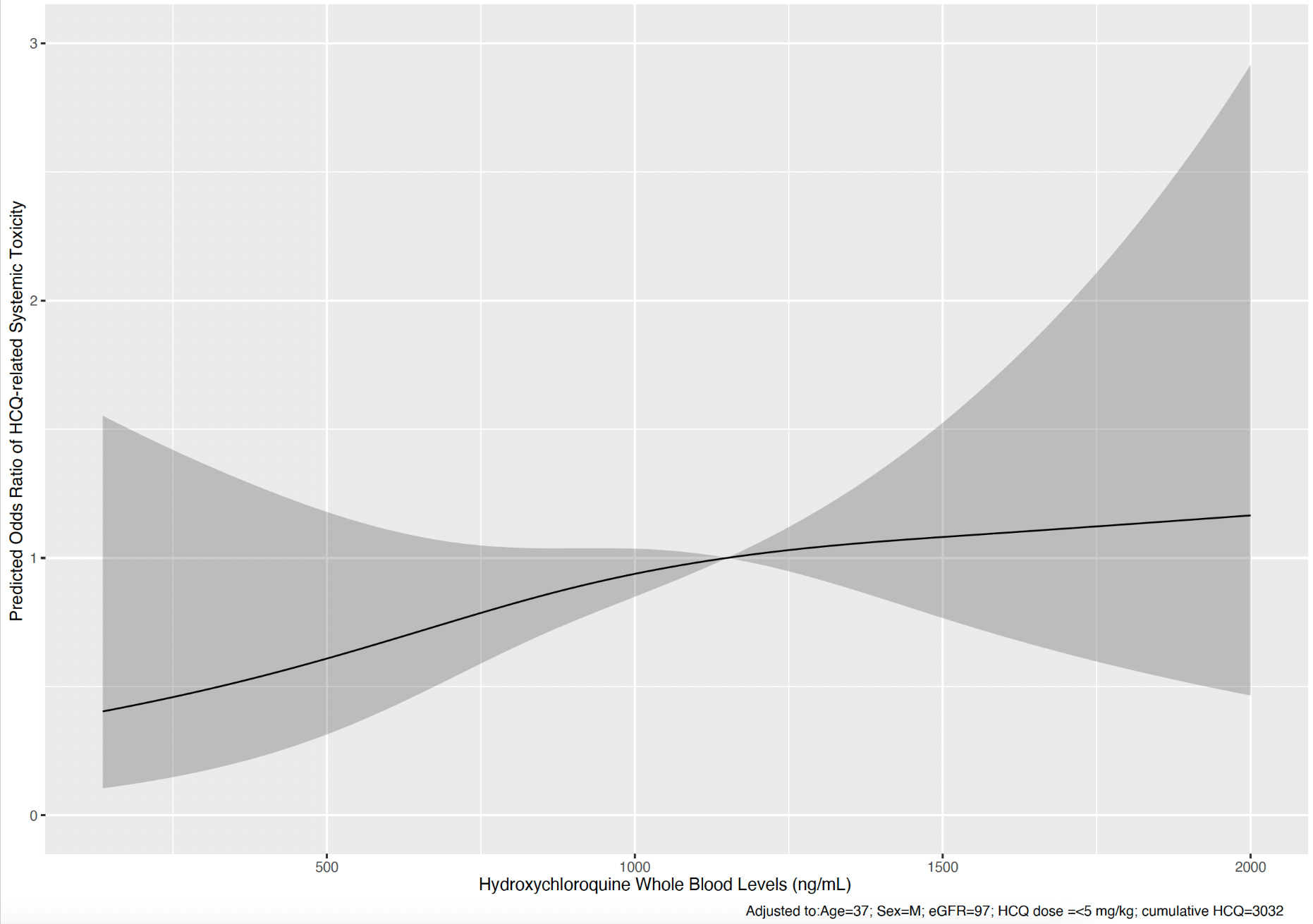
*

**Panel B.**

**
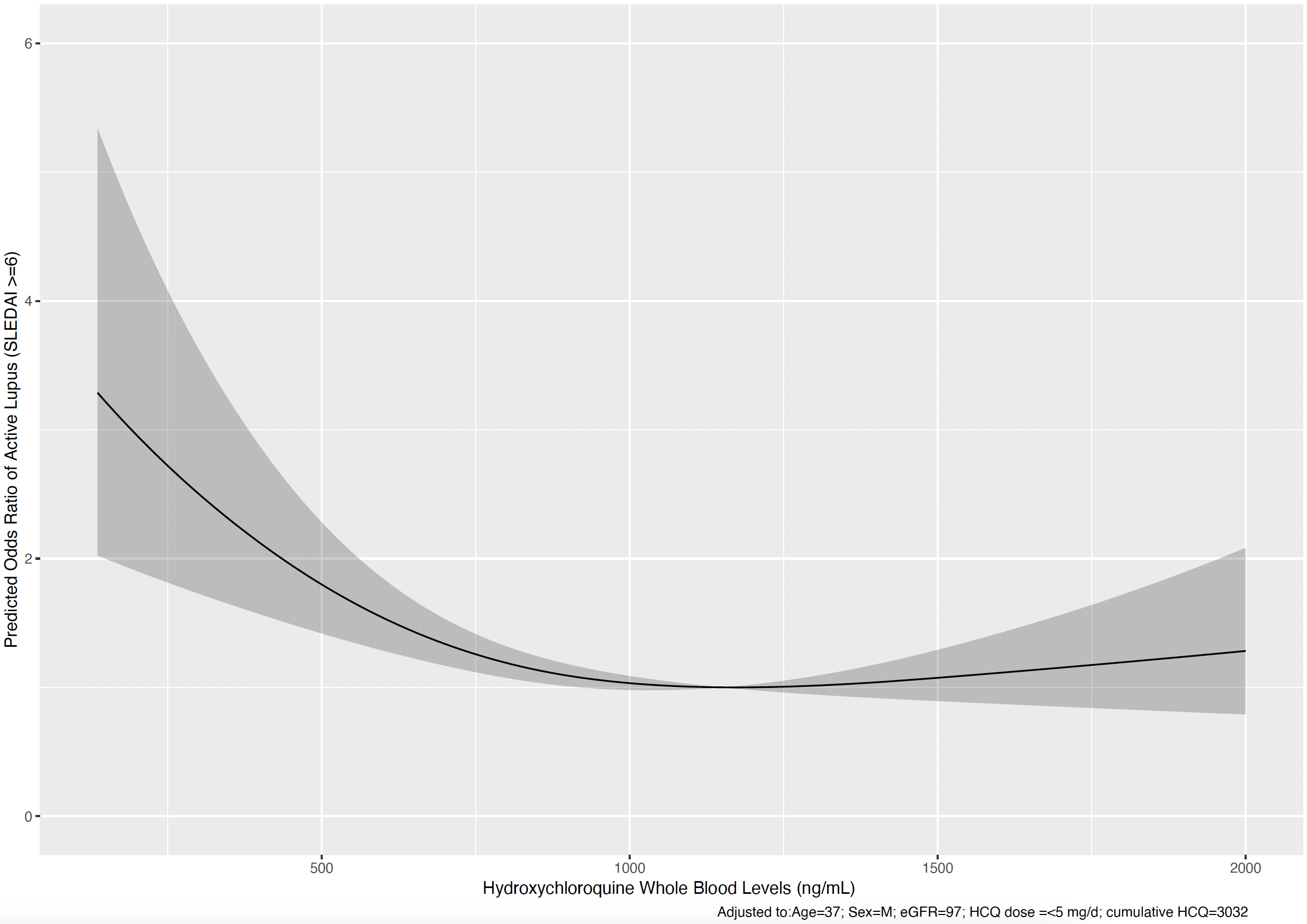
**

**Supplementary Figure 2. Adjusted restricted cubic splines to show associations between HCQ serum levels and toxicity (Panel A), and active SLE (SLEDAI ≥6) (Panel B), in the SLICC cohort (n=602^1^).**

**1:** *58 patients with very low HCQ whole blood levels <200 ng/mL (HCQ serum levels <106 ng/mL) were excluded from analysis*


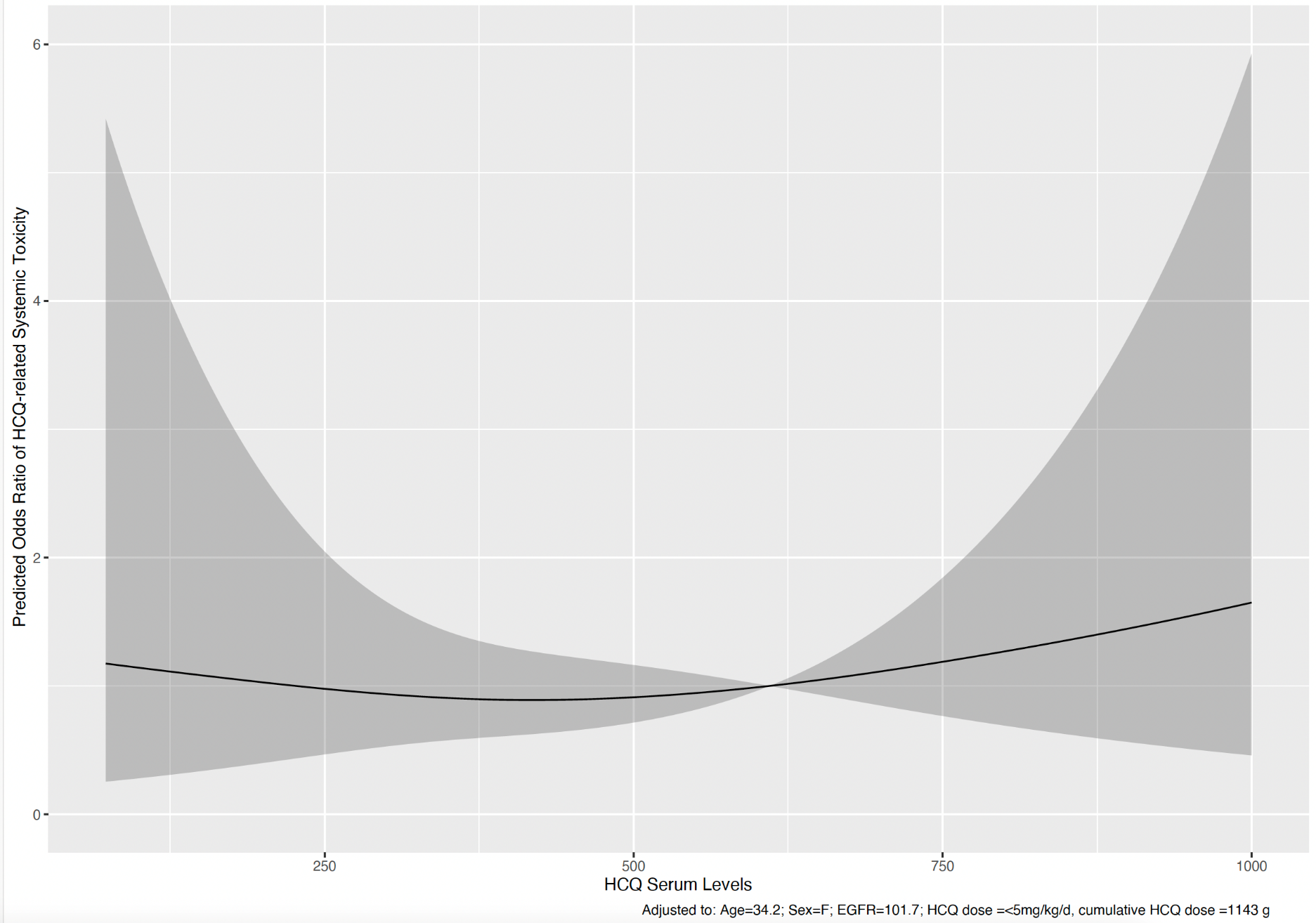


**Panel A.**

**Panel B.**


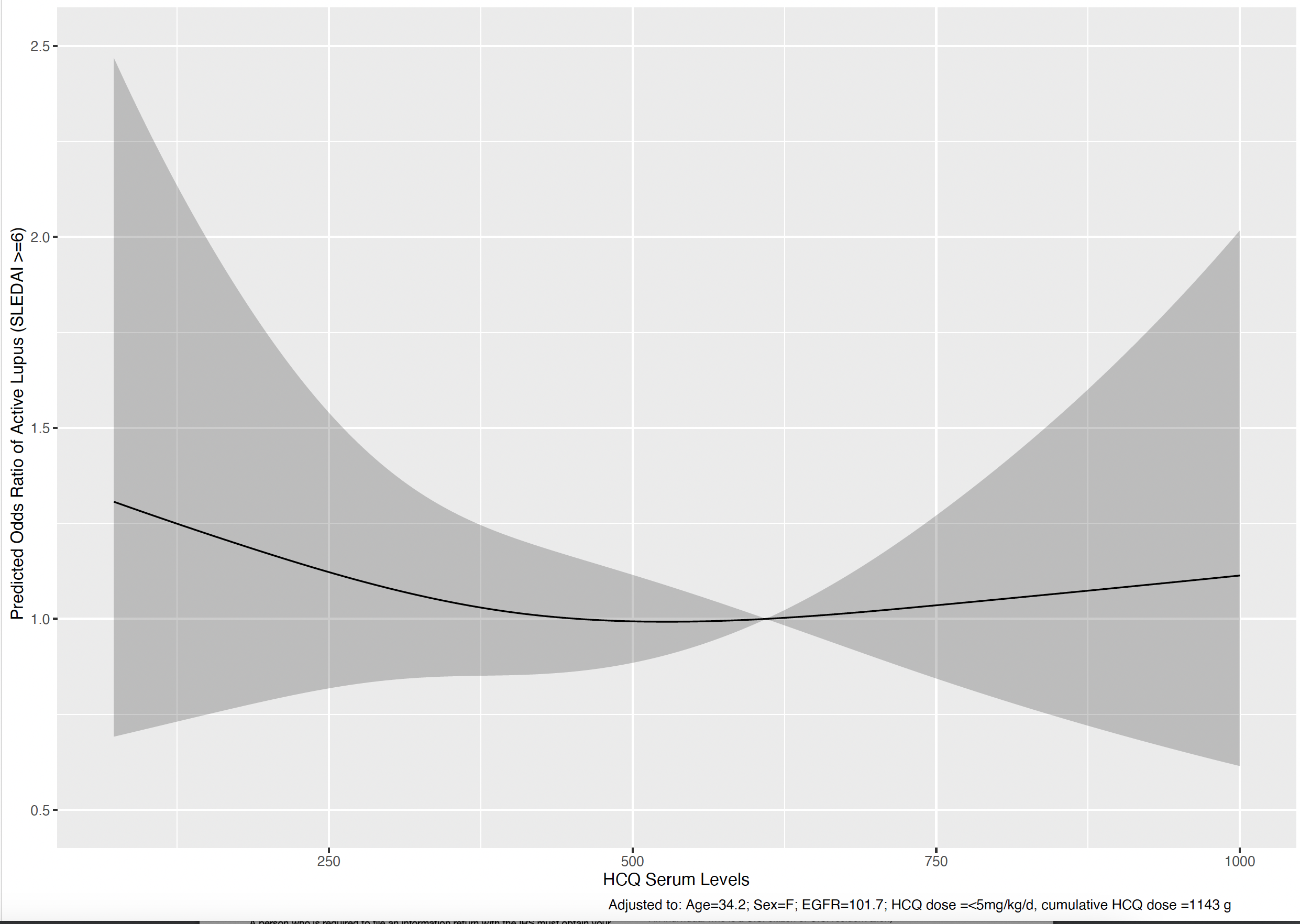


**Supplementary Figure 3.** Frequency plot showing number of patients on weight-based HCQ dosing (≤5mg/kg/day; **Panel A**) and those on >5mg/kg/day HCQ dosing (**Panel B**) with subtherapeutic (<750 ng/mL), therapeutic (750-<1150 ng/mL), and supratherapeutic levels (≥1150 ng/mL)

*
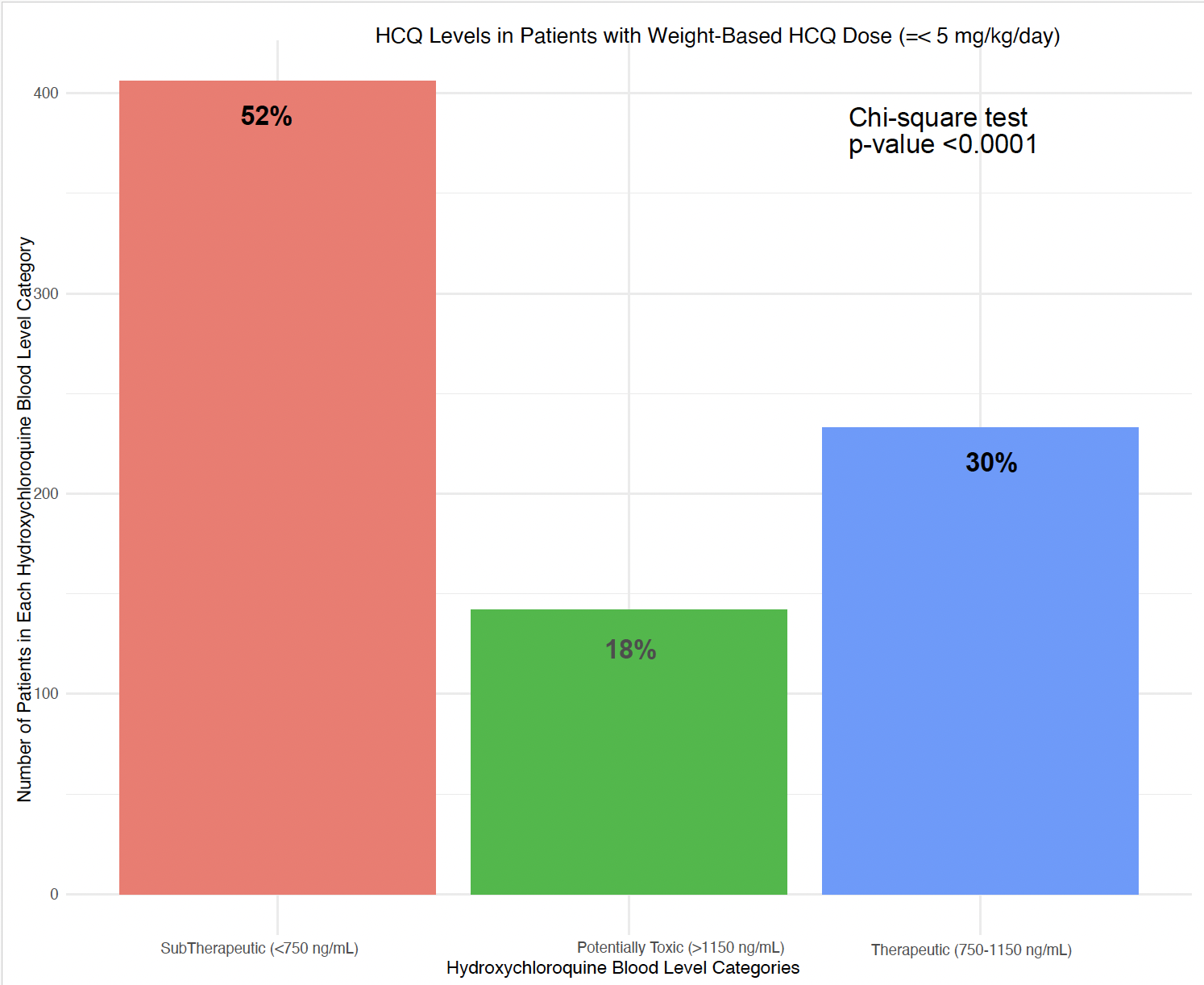
*

**Panel A.**

**
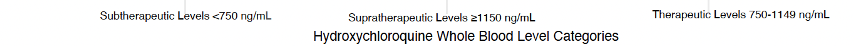
**

**
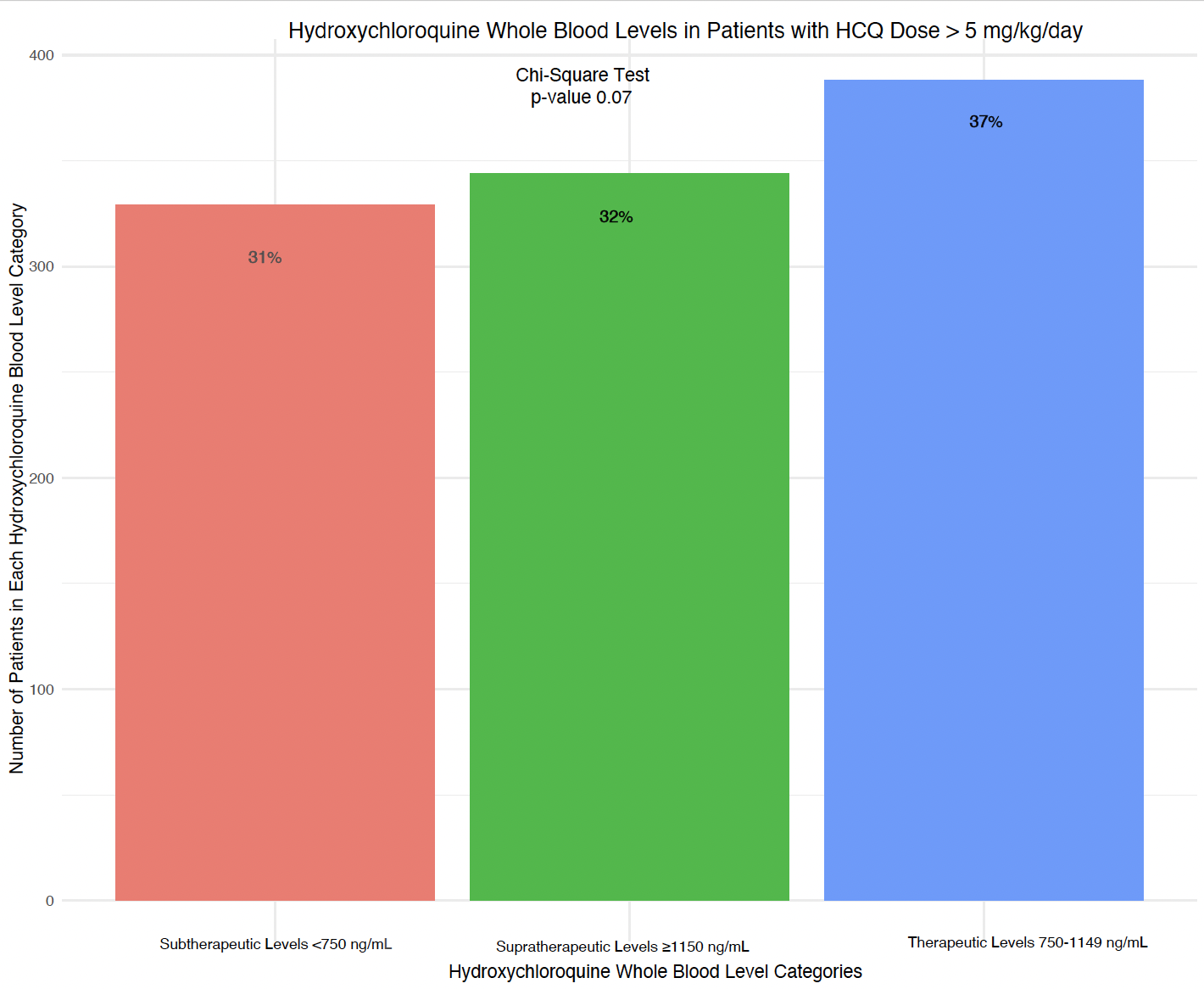
**

**Panel B.**

|  | |
| --- | --- |
| **Predicted change in HCQ**  **Whole Blood levels based on weight-based HCQ dose and by CKD stage** | |
| **Variables** | **Predicted Change in HCQ Whole Blood Levels** |
| HCQ weight based dose per 1 mg/kg/day increase | 66±6 ng/mL |
| CKD stages 1-2 (shown as red line) | ref |
| CKD stage 3a (shown as green line) | 136±59 ng/mL |
| CKD stages 3b-5 (shown as blue line) | 166±59 ng/mL |

*
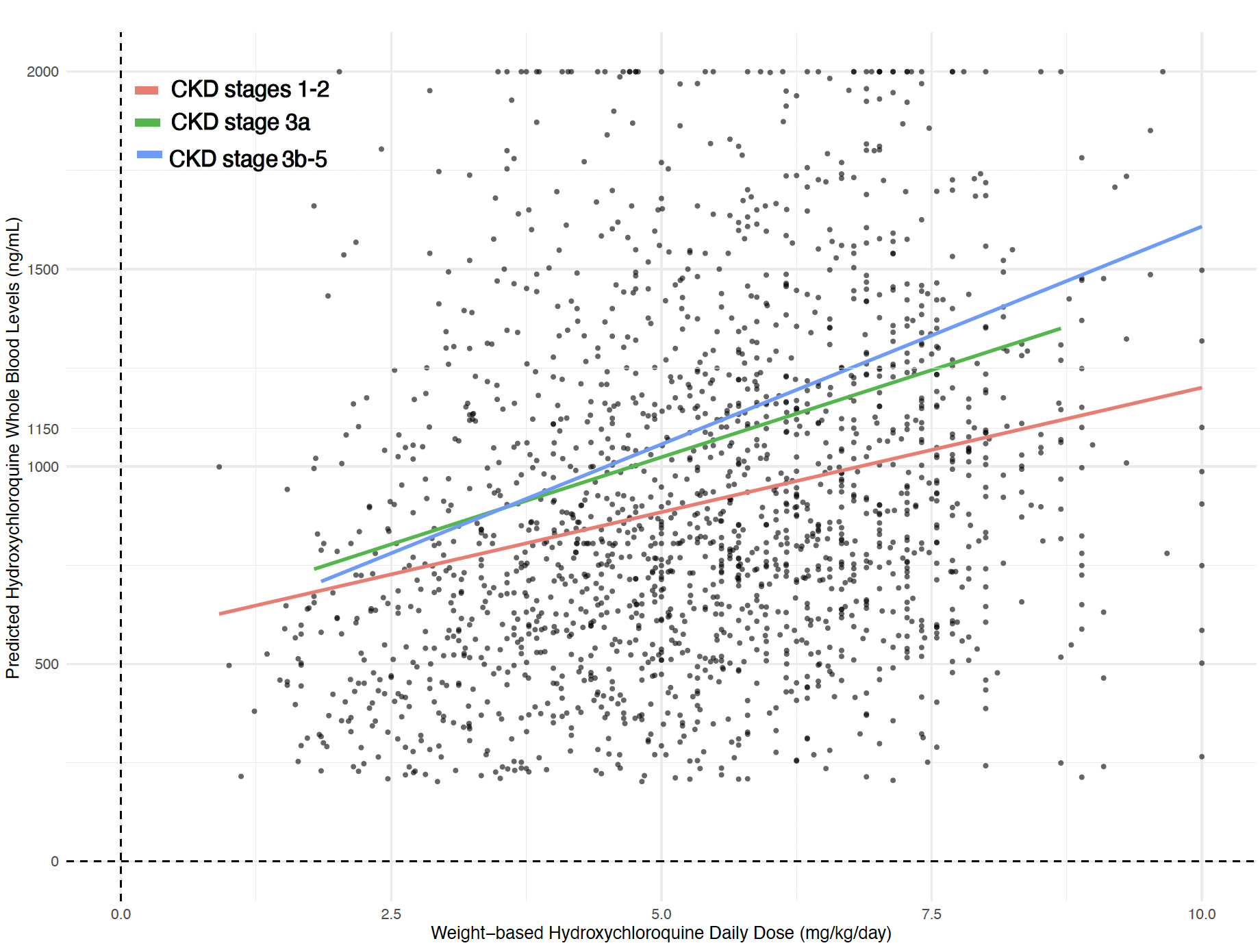
***Supplementary Figure 4: Regression plot showing cross-sectional associations between continuous weight-based HCQ daily dose (in mg/kg/day) and predicted HCQ Whole Blood levels (in ng/mL) by CKD stages: 1-2 (≥60 ml/min/1.73m^2^) vs. 3a (45-59 ml/min/1.73m^2^) vs. 3b-5 (<45 ml/min/1.73m^2^)**
